# Ant-Neointimal Formation Effects of SLC6A6 in Preventing Vascular Smooth Muscle Cell Proliferation and Migration via Wnt/β-Catenin Signaling

**DOI:** 10.3390/ijms24033018

**Published:** 2023-02-03

**Authors:** Zhihua Rong, Fengshi Li, Rui Zhang, Shuai Niu, Xiao Di, Leng Ni, Changwei Liu

**Affiliations:** Department of Vascular Surgery, Peking Union Medical College Hospital, Chinese Academy of Medical Sciences and Peking Union Medical College, Beijing 100730, China

**Keywords:** vascular remolding, neointimal formation, SLC6A6, VSMCs

## Abstract

Vascular smooth muscle cells (VSMCs) play an important role in the pathogenesis of vascular remolding, such as atherosclerosis and restenosis. Solute carrier family 6 member 6 (SLC6A6) is a transmembrane transporter that maintains a variety of physiological functions and is highly expressed in VSMCs. However, its role on VSMCs during neointimal formation remains unknown. In this study, mRNA and protein levels of SLC6A6 were examined using models of VSMC phenotype switching in vivo and in vitro and human artery samples with or without atherosclerosis. SLC6A6 gain- and loss-of-function approaches were performed by adenovirus infection or small interfering RNA (siRNA) transfection, respectively. Reactive oxygen species (ROS), proliferation, migration, and phenotype-related proteins of VSMCs were measured. Vascular stenosis rate and related genes were assessed in a rat vascular balloon injury model overexpressing SLC6A6. SLC6A6 was downregulated in dedifferentiated VSMCs, atherosclerotic vascular tissues, and injured vascular tissues. SLC6A6 suppressed VSMC proliferation and migration, while increasing contractile VSMC proteins. Mechanistically, SLC6A6 overexpression reduced ROS production and inhibited the Wnt/β-catenin pathway. Furthermore, SLC6A6 overexpression suppressed neointimal formation in vivo. Collectively, overexpression of SLC6A6 suppresses neointimal formation by inhibiting VSMC proliferation and migration via Wnt/β-catenin signaling and maintaining the VSMC contractile phenotype.

## 1. Introduction

Atherosclerosis is a chronic arterial disease with high morbidity and mortality rates [1]. Angioplasty is a minimally invasive procedure used to widen blocked arteries in the treatment of atherosclerosis; however, it may lead to neointimal formation, which is characterized by arterial wall thickening. Neointimal formation results in the re-narrowing of the artery, also known as restenosis, which reduces vascular patency [2,3]. Taking femoropopliteal artery disease with stent implantation as an example, nearly 30% to 40% of patients will experience restenosis within 2 years after angioplasty, and these patients will have to undergo reoperation for vascular reconstruction or even amputation [4]. Therefore, it is urgent to investigate the molecular mechanisms underlying neointimal formation to improve the long-term efficacy of angioplasty.

Neointimal formation is a common process in many vascular-remodeling-related diseases, including atherosclerosis and restenosis [5], in which vascular smooth muscle cells (VSMCs) play an important role. VSMCs exhibit phenotypic plasticity: in response to vascular injury, VSMCs change from a contractile phenotype, characterized by low proliferation and migration, to a dedifferentiated synthetic phenotype with high proliferative and migratory abilities and reduced expression of contractile markers, such as the smooth muscle myosin heavy chain (SMMHC), smooth muscle α-actin (α-SMA), and transgelin (SM22α) [6]. The proliferation and migration of synthetic VSMCs to the intima, accompanied by high extracellular matrix (ECM) deposition, lead to neointimal formation [7,8].

Solute carrier family 6 member 6 (SLC6A6) is a multi-pass membrane protein that regulates cell proliferation, differentiation, calcification, and apoptosis [9,10,11,12]. SLC6A6 loss of function induces various diseases, including retinal degeneration [13,14], sarcopenia [15], diabetic nephropathy [16], and hyperammonemia [17]. SLC6A6 is also associated with cardiovascular diseases, such as dilated cardiomyopathy [18], acute myocardial infarction [19], and ventricular remodeling [20]. A functional aspect of SLC6A6 is that it actively transports taurine into cells to maintain high intracellular concentrations [21]. Taurine is a non-protein amino acid that has significant antioxidative [22,23], anti-inflammatory [24], and anti-aging properties [25]. Furthermore, previous studies have confirmed that taurine can suppress VSMC proliferation [9], neointimal formation [26], and hypertension [27] and has anti-atherogenic effects [28]. Notably, SLC6A6, a transporter of taurine, is highly expressed in VSMCs [29]; however, its role in VSMCs is unclear.

In this study, we demonstrate that SLC6A6 expression is reduced in dedifferentiated VSMCs, atherosclerotic arteries, and vascular tissues after injury. SLC6A6 overexpression reduced neointimal formation by maintaining the VSMC contractile phenotype and inhibiting VSMC proliferation and migration, at least in part, through the Wnt/β-catenin pathway.

## 2. Materials and Methods

### 2.1. Animal Experiments

Rat carotid balloon injury model was performed as described previously [30]. Briefly, male Sprague Dawley rats weighing between 250 and 300 g were purchased from Beijing Vital River Laboratory. After one week of adaptive rearing in an SPF environment with 60 to 65% humidity and 22 to 25 °C temperature, rats were anesthetized by intraperitoneal injection of pentobarbital sodium (40 mg/kg). A midline incision was made in the neck, subcutaneous tissue and fascia were separated, and the left carotid artery was carefully exposed. The left external carotid artery was ligated above the superior thyroid artery with 5-0 silk thread, and a 2F Fogarty balloon catheter (Edward Lifesciences, CA, USA) was inducted into the left external carotid artery, and then 0.2 mL of saline was used to distend the balloon, and the catheter was stably rotated and withdrawn to the carotid bifurcation. This procedure was repeated three times to ensure complete endothelial denudation. The sham group was subjected to a similar operation without balloon injury. The carotid arteries were collected at 7, 14, and 28 days after the operation for following experiments.

Eighteen rats were divided into three groups: sham operation group, injured group treated with adenoviral vectors expressing SLC6A6 (Ad-SLC6A6) or control green fluorescent protein (Ad-GFP), produced by Hanbio Biotechnology (Wuhan, China). After vascular injury, 200 μL of Ad-SLC6A6 or Ad-GFP (1 × 10^10^ PFU/mL) was infused into the injured segment of the common carotid artery via a catheter and incubated for 30 min at room temperature to ensure sufficient infection. The catheter was removed, the external carotid artery was ligated, and the incision was carefully closed. Carotid arteries were collected 14 days after the operation for follow-up experiments.

### 2.2. Cell Culture

Primary VSMCs were obtained from the thoracic aorta of Sprague Dawley rats (160–200 g) as previously described [30]. Briefly, the adventitia and endothelium of fresh vascular tissue were removed, the medial layer was cut into small pieces and cultured in DMEM (Gibco, MI, USA) containing 20% FBS (fetal bovine serum, Gibco), and the medium was replaced with a medium containing 10% FBS when VSMCs sprouted from the tissue block. The purity of VSMCs was assessed using immunofluorescence staining for VSMCs’ specific marker, α-SMA (1:200; Abcam, Cambridge, UK). Cells passaged 3–6 times were used for all experiments. VSMCs were serum-starved for 24 h before treatment with 20% FBS or platelet-derived growth factor-BB (PDGF-BB; R&D Systems, MN, USA).

### 2.3. SLC6A6 Knockdown or Overexpression In Vitro

Small interfering RNAs (siRNA) against SLC6A6 (si-SLC6A6) and a negative control siRNA (NC) were produced by RiboBio (Guangzhou, China). VSMCs were transfected with 100 nmol/L si-SLC6A6 or NC using RNAiMax (Invitrogen, CA, USA). As described in the animal section, adenovirus vectors (AD-SLC6A6, 100 multiplicities of infection (MOI)) were used to overexpress SLC6A6 in VSMCs, and the control group was transfected with green fluorescent protein (Ad-GFP, 100 MOI). After 24 h, the culture medium was replaced with fresh medium with or without PDGF-BB (50 ng/mL) for an additional 24 h.

### 2.4. RNA Extraction and Quantitative Real-Time Polymerase Chain Reaction (qRT-PCR)

Total RNA was extracted from VSMCs or vascular tissues following the TRIzol extraction protocol (Invitrogen). Equal amounts of RNA were reverse transcribed into cDNA using the Prime Script RT Reagent Kit (TAKARA, Dalian, China). qRT-PCR amplification was performed with the THUNDERBIRD^®^ Next SYBR qPCR Mix (TOYOBO, Osaka, Japan) on a CFX Opus 96 system (Bio-Rad, CA, USA). The mRNA levels were normalized to those of GAPDH. Data were analyzed using CFX Maestro qPCR analysis software version (V2.3). The primer sequences are listed in Supplementary Table S1.

### 2.5. Western Blotting

Western blotting experiments were performed as previously described [30]. Briefly, total protein of VSMCs and vascular tissue was extracted with RIPA lysis buffer (Beyotime, Shanghai, China) and protease inhibitor (Thermo Fisher Scientific, CA, USA) mixture. The protein concentration was measured using a BCA kit (Thermo Fisher Scientific, CA, USA). Equal amounts of protein were separated by 12.5% SDS-PAGE and then transferred to PVDF membrane (MilliporeSigma, MA, USA), blocked in 5% milk for 1 h at room temperature. Then, they were incubated with the following primary antibodies at 4 °C overnight: anti-SLC6A6 (1:1000; Cat#ab236898; Abcam, MA, USA), anti-α-SMA (1:10,000; Cat#55135-1-AP; Proteintech, Wuhan, China), anti-SM22α (1:10,000; Cat# ab14106; Abcam), anti-SMMHC (1:1000; Cat#21404-1AP; Proteintech), anti-CNN1 (1:1000; Cat#17819; Cell Signaling Technology, MA, USA), anti-cyclin D1 (1:10000; Cat#ab134175; Abcam, MA, USA), anti-β-catenin (1:1000; Cat# ab32572; Abcam, MA, USA), and anti-β-actin (1:1000; Cat# 66009-1-Ig; Proteintech,Wuhan, China). The membranes were then incubated with anti-mouse or anti-rabbit HRP-conjugated antibodies (1:10,000; EasyBio, Beijing, China). Images were acquired using an optical scanner and analyzed using ImageJ software ( *ImageJ software* v1.6.0, MD, USA) [31].

### 2.6. VSMC Proliferation Assay

Cell-Light EdU Apollo567 In Vitro Kit (RiboBio, Guangzhou, China) and CCK-8 kit (Dojindo, Shanghai, China) were used to detect the proliferation of VSMCs following the manufacturers’ protocols. Briefly, for EdU assay, proliferating cells were labeled with EdU, and Hoechst (RiboBio, Guangzhou, China) was used to label all cells. Five fields were randomly selected to take pictures under a fluorescence microscope (Nikon, Tokyo, Japan) and analyzed using ImageJ software. For CCK-8 assay, treated VSMCs were seeded in 96-well plates at 8 × 10^3^ cells/well for 24 h and then incubated with CCK-8 solution at 37 °C for 2 h. Absorbance at 450 nm was measured using a microplate reader (Bio-Tek, CA, USA).

### 2.7. VSMC Migration Assay

Cells migration was measured using a wound-healing and migration model in Culture-Inserts (ibidi GmbH, Munich, Germany). Treated VSMCs (7 × 10^3^; 70 μL volume) were seeded in each well of the Culture-Inserts and incubated for 24 h. Culture-Inserts were removed the following day and replaced with a serum-free medium. Photographs were taken every 24 h using a microscope (Nikon, Tokyo, Japan), and the migration area was analyzed using ImageJ software.

Using Transwell assay, treated VSMCs were resuspended in serum-free medium, and 200 μL of cells suspension (2 × 10^4^ cells) were added on the upper layer of the Transwell chamber (24-well plate, 0.8 mm pores, Corning, NY, USA), and medium containing 20% FBS was added to lower layer and then incubated at 37 °C for 24 h. Following day, the unmigrated VSMCs on the upper layer were wiped off with a cotton swab, and the migrated VSMCs on the lower layer were fixed with 4% paraformaldehyde for 30 min at room temperature and stained with 0.1% crystal violet. Five fields of view were randomly selected under a microscope to take pictures, and ImageJ software was used for data analysis.

### 2.8. ROS Mearsurement

VSMCs intracellular ROS was measured by dihydroethidium (DHE) (Sigma, MO, USA) according to the manufacturer’s protocol. Briefly, treated VSMCs were incubated with DHE (10 μM) in a cell incubator at 37 °C for 1 h, and then ROS levels were observed in five arbitrarily selected fields under a fluorescence microscope.

### 2.9. Human Artery Samples Collection

Healthy and diseased human arterial samples were collected from patients at Peking Union Medical College Hospital, China, between November 2020 and May 2022. Healthy femoral arterial specimens were acquired from patients without peripheral artery disease (e.g., trauma) (Healthy, *n* = 4). Diseased femoral arterial samples were collected from patients undergoing bypass grafting or amputation due to lower limb arteriosclerosis obliterans (AS, *n* = 4). Patient baseline characteristics are listed in Supplementary Table S2.

### 2.10. Histological and Morphometric Analyses

Vascular tissues were fixed with 4% paraformaldehyde solution (Solarbio, Beijing, China) and embedded in paraffin after dehydration. Sections were sliced at a thickness of 4 µm, some were stained with hematoxylin and eosin (H&E), and some were used for subsequent immunohistochemistry or immunofluorescence experiments. Images were captured using a digital slice scanner (KFBIO, Ningbo, China). ImageJ software was used to analyze the intimal area and the medial area.

### 2.11. Immunohistochemical and Immunofluorescence Staining

For cellular immunofluorescence, VSMCs were fixed with pre-cooled methanol for 5 min, blocked with 10% goat serum, and incubated with the following primary antibodies at 4 °C overnight: anti-SLC6A6 (1:50; Abcam, MA, USA) and anti-α-SMA (1:100; Proteintech, Wuhan, China). Paraffin sections were subjected to routine dewaxing and antigen retrieval. The sections were blocked in 10% goat serum at room temperature for 1 h and incubated with the following primary antibodies at 4 °C overnight: anti-SLC6A6 (1:100; Abcam, MA, USA), anti-α-SMA (1:200; Proteintech, Wuhan, China), anti-cyclin D1 (1:50; Abcam, MA, USA), and anti-PCNA (1:100; Proteintech, Wuhan, China). Immunohistochemical staining was performed with a DAB kit (ZSGB-BIO, Beijing, China) according to the instructions. Images were obtained using a digital slice scanner (KFBIO, Ningbo, China). For immunofluorescence staining, cells or vascular samples were incubated with the fluorescent secondary antibodies Alexa Flour 488-conjugated goat anti-mouse (1:200, Proteintech, Wuhan, China) and Alexa Flour 594-conjugated goat anti-rabbit IgG (1:200, Proteintech, Wuhan, China) for 1 h at room temperature. Nuclei were stained with DAPI (Solarbio, Beijing, China). Fluorescent images were obtained using a confocal laser microscope (Nikon, A1R, Tokyo, Japan) [32].

### 2.12. Statistical Analysis

Data were analyzed using GraphPad Prism 9.3 (*GraphPad* Software, San Diego, CA, USA) and are presented as the mean ± SD. Shapiro–Wilk test and Levene’s test were used to assess the normality and homogeneity of data, respectively. Comparisons between two groups were performed using Student’s *t*-test, and comparisons between ≥3 groups were performed using one-way analysis of variance (ANOVA). At least three independent biological replicates were used for each experiment. Statistical significance was set as *p* < 0.05.

## 3. Results

### 3.1. SLC6A6 Decreased during VSMCs Dedifferentiation

**Figure 1:**
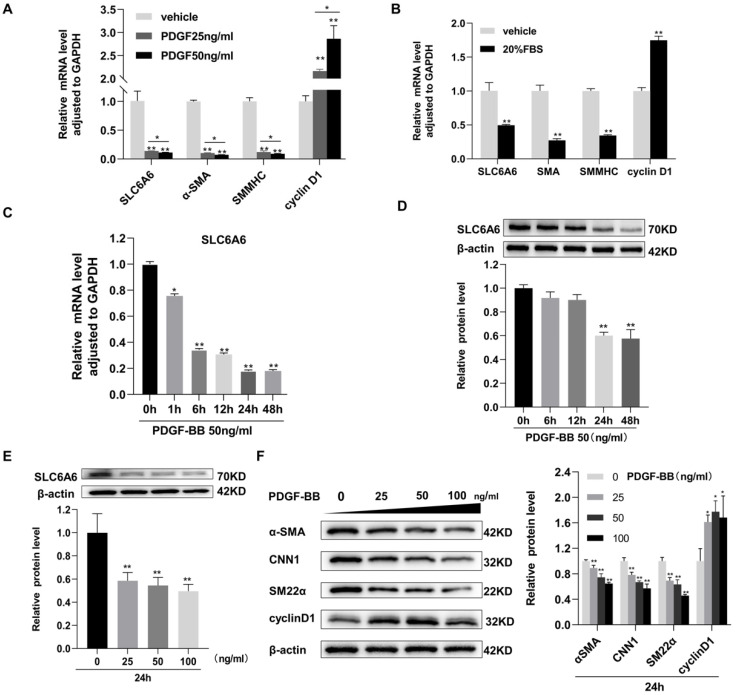

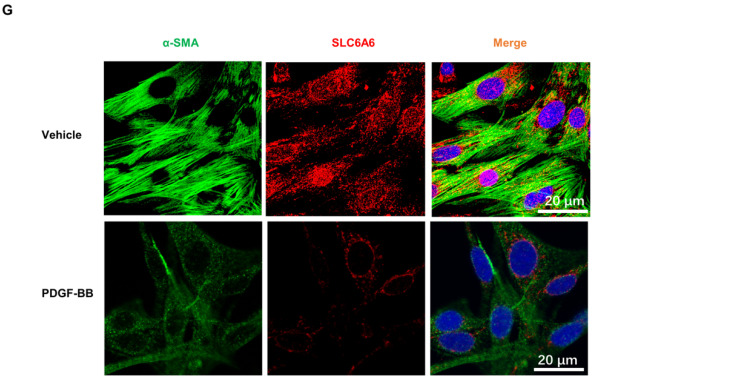
SLC6A6 was downregulated during VSMC dedifferentiation. (**A**,**B**) Quantitative real-time polymerase chain reaction (qRT-PCR) showing the mRNA levels of SLC6A6, VSMC-specific genes (α-SMA and SMMHC), and cyclin D1. VSMCs were stimulated with PDGF-BB (25 ng/mL, 50 ng/mL) or 20% FBS. Data were normalized to GAPDH. N = 3; * p < 0.05, ** *p* < 0.01 vs. vehicle. Data are represented as mean ± SD. (**C**,**D**) qRT-PCR and Western blot analyses showing the time-dependent changes in SLC6A6 mRNA and protein levels after PDGF-BB stimulation (50 ng/mL). Data were normalized to GAPDH (PCR) and β-actin (Western blotting). N = 3; * p < 0.05, ** *p* < 0.01 vs. 0 h. Data are represented as mean ± SD. (**E**,**F**) Concentration-dependent changes in SLC6A6 and α-SMA, CNN1, SM22α, and cyclin D1 protein levels after 24 h of PDGF-BB stimulation (0, 25, 50, and 100 ng/mL). N = 3; * p < 0.05, ** *p* < 0.01 vs. 0 ng/mL. Data are represented as mean ± SD. (**G**) Representative images of dual immunofluorescence staining of SLC6A6 (red) and α-SMA (green) in vehicle (PBS) and PDGF-BB (50 ng/mL)-stimulated VSMCs, scale bar: 20 µm (600×).

We first investigated SLC6A6 expression during VSMC dedifferentiation. Dedifferentiated VSMCs were induced by stimulation with different concentrations of PDGF-BB (25 ng/mL, 50 ng/mL, and 100 ng/mL) or 20% FBS. The mRNA level of SLC6A6 was dramatically decreased under PDGF-BB (25 ng/mL and 50 ng/mL) or 20% FBS stimulation, accompanied with the depression of VSMC-specific genes, α-SMA and SMMHC, while cell proliferation marker cyclinD1 was increased (Figure 1A,B). Moreover, the downregulation mRNA and protein levels of SLC6A6 were time-dependent with PDGF-BB stimulation within 48 h (Figure 1C,D). The protein level of SLC6A6 decreased progressively with the increase of PDGF-BB concentration, which was consistent with VSMC contractile phenotype proteins (α-SMA, CNN1, and SM22α) but contrary to cyclinD1 (Figure 1E,F). Cell immunofluorescence analysis further confirmed that SLC6A6 was downregulated after PDGF-BB-induced VSMC dedifferentiation (Figure 1G). These data imply that SLC6A6 may be involved in VSMC dedifferentiation.

### 3.2. SLC6A6 Downregulated during Neointimal Formation

**Figure 2:**
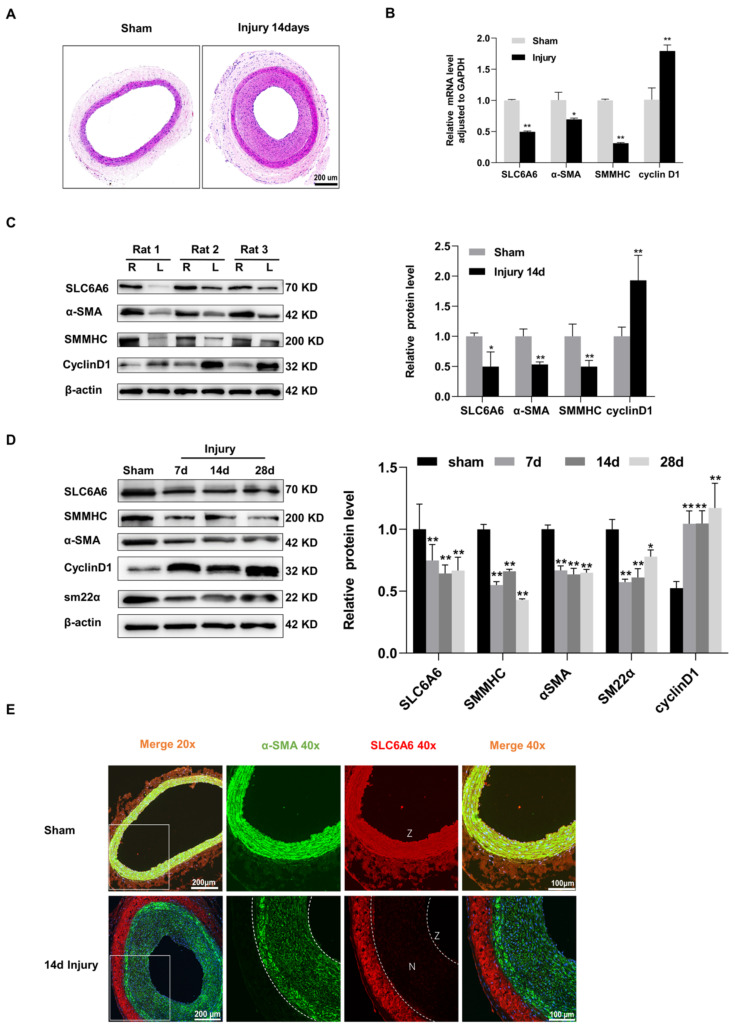
SLC6A6 was downregulated during neointimal formation. (**A**) Representative HE of sham and injured carotid arteries in rats. Scale bar: 200 µm (100×). (**B**) qRT-PCR analysis showing mRNA (SLC6A6, α-SMA, SMMHC, cyclin D1) expression levels in sham and injured carotid arteries. Data are normalized to GAPDH; N = 3; * p < 0.05, ** *p* < 0.01 vs. sham. Data are represented as mean ± SD. (**C**) Western blot analysis of SLC6A6 and contractile VSMC-specific proteins (SMMHC, α-SMA) and cell proliferation marker cyclin D1 in sham (right, R) and injured (left, F) carotid arteries. Data are normalized to β-actin; N = 3; * p < 0.05, ** *p* < 0.01 vs. sham. Data are represented as mean ± SD. (**D**) Protein expression levels of SLC6A6, SMMHC, α-SMA, SM22α, and cyclin D1 in carotid arteries at 7, 14, and 28 days post injury. Data are normalized to β-actin; N = 3; * p < 0.05, ** *p* < 0.01 vs. sham. Data are represented as mean ± SD. (**E**) Representative images of dual immunofluorescence staining of SLC6A6 (red) and α-SMA (green) in sham and injured carotid arteries. N, neointimal; Z, zoom of vascular tissue. Scale bar: 200 µm (200×, left); scale bar: 100 µm (400×, right).

Neointimal formation is the main pathological feature of vascular remodeling. To investigate the involvement of SLC6A6 in neointimal formation, we established a balloon-injured rat carotid artery model, which is a classic model of arterial neointimal formation (Figure 2A). Fourteen days after injury, qRT-PCR and Western blotting showed that mRNA and protein levels of SLC6A6 were decreased in injured vascular tissues compared to uninjured vascular tissues. Along with the downregulation of SLC6A6 after vascular injury, VSMC contractile markers (α-SMA, SMMHC) were downregulated, whereas the proliferation marker cyclin D1 was increased (Figure 2B,C). We observed similar results at 7, 14, and 28 days after injury (Figure 2D). Immunofluorescence analysis further confirmed that SLC6A6 and α-SMA were predominantly colocalized to VSMCs in the media of uninjured vascular tissues, whereas SLC6A6 was barely expressed in the neointima after injury (Figure 2E). These data suggest that SLC6A6 may play a role in neointimal formation.

### 3.3. SLC6A6 Decreased in Atherosclerosis

**Figure 3:**
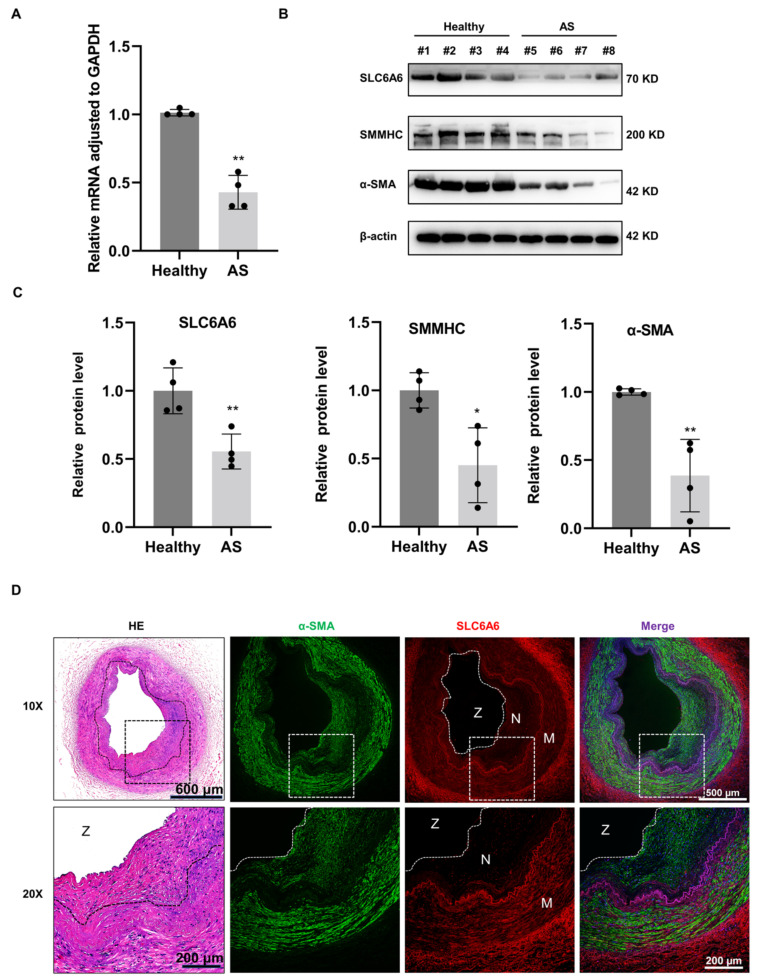
SLC6A6 expression was decreased in atherosclerotic arteries. (**A**) qRT-PCR analysis showing SLC6A6 mRNA expression levels in healthy and atherosclerotic arteries (AS). Data are normalized to GAPDH; N = 4; * p < 0.05, ** *p* < 0.01 vs. healthy. Data are represented as mean ± SD. Each dot represents an individual sample. (**B**) and (**C**) Western blot analysis of SLC6A6, SMMHC, and α-SMA expression levels in healthy human and AS arteries. Data are normalized to β-actin; N = 4; * p < 0.05, ** *p* < 0.01 vs. healthy. Data are represented as mean ± SD. Each black dot represents an individual sample. (**D**) Representative images of dual immunofluorescence staining of SLC6A6 (red) and α-SMA (green) in atherosclerotic arteries. M, medial; N, neointimal; Z, zoom of vascular tissue. N = 4 per group; scale bar: 600 µm (80× upper), scale bar: 500 µm (100×), scale bar: 200 µm (200× lower).

To translate our findings from rats to humans, we examined the expression of SLC6A6 in human femoral arteries from patients with or without atherosclerosis. Compared with healthy femoral arteries, both SLC6A6 mRNA and protein levels were decreased in atherosclerotic vascular tissues (Figure 3A,B). Moreover, the downregulation of SLC6A6 protein in atherosclerotic tissues is accompanied with the depression of VSMC contractile proteins (SMMHC, α-SMA) compared to normal arteries (Figure 3B,C). Immunofluorescence analysis demonstrated that SLC6A6 was expressed in the medial layer and adventitia but not in the neointimal layer of atherosclerotic arteries (Figure 3D). These results were consistent with animal experiments, suggesting that SLC6A6 plays an important role in the process of neointimal formation of atherosclerosis.

### 3.4. SLC6A6 Knockdown Promoted VSMCs Dedifferentiation, Proliferation, and Migration

**Figure 4:**
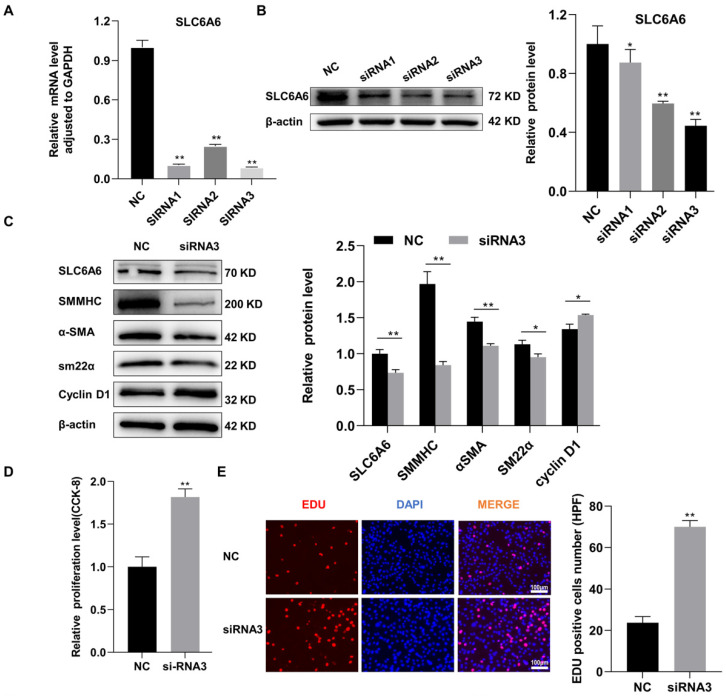

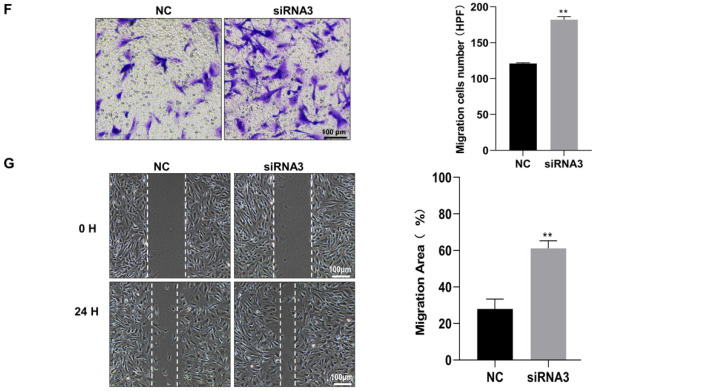
SLC6A6 knockdown induced VSMC dedifferentiation, proliferation, and migration. (**A**) qRT-PCR and (**B**) Western blot analysis of SLC6A6 mRNA and protein levels in VSMCs transfected with negative control (NC) or SLC6A6 siRNAs (siRNA 1, siRNA 2, or siRNA 3; 100nmol/L), respectively. Data are normalized to GAPDH for qRT-PCR, data are normalized to β-actin for Western blotting; N = 3; * p < 0.05, ** *p* < 0.01 vs. NC. Data are represented as mean ± SD. (**C**) Western blot analysis of SLC6A6, SMMHC, α-SMA, SM22α, and cyclin D1 after siRNA3 transfection. Data are normalized to β-actin; N = 3; * p < 0.05, ** *p* < 0.01 vs. NC. Data are represented as mean ± SD. (**D**) CCK-8 and (**E**) EdU assays showing VSMC proliferation after siRNA3 transfection. Scale bar: 100 µm (200×). N = 3; * p < 0.05, ** *p* < 0.01 vs. NC. Data are represented as mean ± SD. (**F**) Transwell assays and (**G**) wound-healing assays showing VSMC migration after siRNA3 transfection for 24 h. N = 3; scale bar: 100 µm (100×). HPF, high power field.; N = 3; * p < 0.05, ** *p* < 0.01 vs. NC. Data are represented as mean ± SD.

To investigate the function of SLC6A6 in VSMCs, three candidate siRNAs (siRNA1, siRNA2, and siRNA3) targeting SLC6A6 were used to downregulate SLC6A6 expression in vitro. siRNA efficiency was verified by qRT-PCR and Western blotting, which demonstrated that siRNA3 markedly decreased SLC6A6 mRNA and protein levels compared to the negative control (NC) (Figure 4A,B). Therefore, we selected siRNA3 for subsequent studies. SLC6A6 knockdown resulted in the downregulation of contractile VSMC-specific proteins (SMMHC, α-SMA, and SM22α) while upregulating cyclin D1, suggesting the transition of VSMCs to a proliferative state (Figure 4C). The proliferation of VSMCs was assessed using CCK-8 and EdU assays, and both revealed that SLC6A6 downregulation promoted VSMC proliferation (Figure 4D,E). The migration of VSMCs was measured using Transwell and scratch-wound-healing assays, which revealed that SLC6A6 knockdown enhanced VSMC migration (Figure 4F,G). Collectively, these results indicate that SLC6A6 downregulation promotes VSMC dedifferentiation, proliferation, and migration.

### 3.5. SLC6A6 Overexpression Reduced PDGF-BB-Induced VSMC Proliferation and Migration and Dedifferentiation

**Figure 5:**
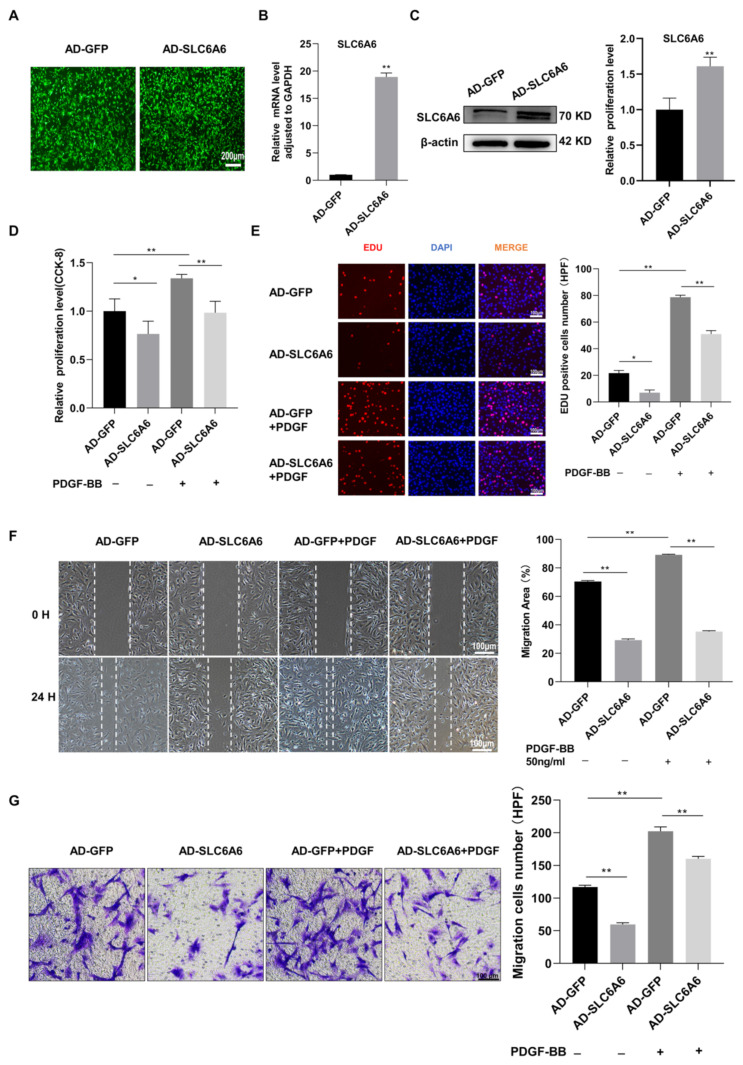
SLC6A6 overexpression reduced PDGF-BB-induced VSMC proliferation and migration. (**A**) Efficiency of adenovirus infection in VSMCs was observed under fluorescence microscope. Scale bar: 200 µm (200×). (**B**) qRT-PCR and (**C**) Western blot analyses showing SLC6A6 mRNA and protein levels after infecting VSMCs with AD-GFP or AD-SLC6A6; N = 3; * p < 0.05, ** *p* < 0.01 vs. AD-GFP. Data are represented as mean ± SD. (**D**) CCK-8 assays and (**E**) EdU assays showing VSMC proliferation after overexpressing SLC6A6 for 24 h with or without PDGF-BB (50 ng/mL) for another 24 h, respectively. Scale bar: 100 µm (200×). N = 3; * p < 0.05, ** *p* < 0.01. Data are represented as mean ± SD. (**F**,**G**) Wound-healing and Transwell assays showing VSMC migration after overexpressing SLC6A6 for 24 h with or without PDGF-BB (50 ng/mL) for another 24 h. Scale bar: 100 µm (100×). HPF, high power field. N = 3; * p < 0.05, ** *p* < 0.01. Data are represented as mean ± SD.

**Figure 6:**
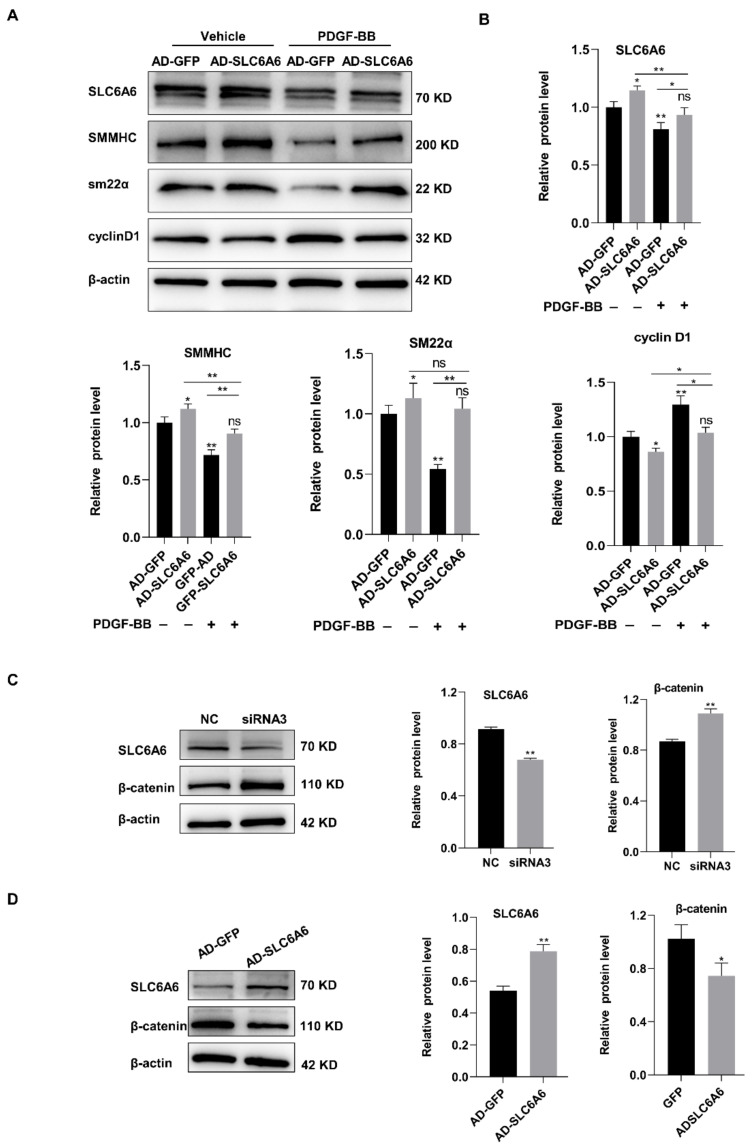
SLC6A6 regulated VSMC functions via β-catenin signaling. (**A**,**B**) Western blot analysis of SLC6A6 and contractile VSMC-specific proteins (SMMHC, SM22α) and cells proliferation marker cyclin D1 after infecting VSMCs with AD-GFP or AD-SLC6A6 for 24 h with or without PDGF-BB (50 ng/mL) for another 24 h, respectively. Data are normalized to β-actin; N = 3; * p < 0.05, ** *p* < 0.01; ns, not significant; data are represented as mean ± SD. (**C**) Western blot analysis of SLC6A6 and β-catenin proteins in VSMCs after transfection with normal control (NC) or siRNA 3 (siRNA-SLC6A6). Data are normalized to β-actin; N = 3; * p < 0.05, ** *p* < 0.01 vs. NC; data are represented as mean ± SD. (**D**) Western blot analysis of SLC6A6 and β-catenin protein levels in VSMCs after infection with AD-GFP or AD-SLC6A6. Data are normalized to β-actin; N = 3; * p < 0.05, ** *p* < 0.01 vs. AD-GFP; data are represented as mean ± SD.

To further investigate the functional effects of SLC6A6 on VSMC proliferation and migration, we overexpressed SLC6A6 in vitro using adenoviral vectors. Adenovirus infection efficiency was greater than 90% (Figure 5A). SLC6A6 mRNA and protein levels were verified by qRT-PCR and Western blotting, respectively (Figure 5B,C). VSMC proliferation was assessed using CCK-8 and EdU assays, and PDGF-BB induced VSMC proliferation and migration, while SLC6A6 overexpression effectively reduced the proliferation of VSMCs (Figure 5D,E). We also measured VSMC migration using Transwell and scratch-wound-healing assays, which revealed that SLC6A6 overexpression reduced the migratory ability of VSMCs (Figure 5F,G). The abilities of proliferation and migration are closely related to VSMC phenotypic switching; therefore, we further explored the role of SLC6A6 in VSMC phenotypic switching by analyzing the expression of contractile VSMC-specific proteins and proliferation markers after SLC6A6 overexpression in vitro. In contrast to SLC6A6 knockdown, SLC6A6 overexpression resulted in the upregulation of contractile proteins (SMMHC and SM22α) concomitant with the downregulation of cyclin D1 (Figure 6A,B). Collectively, these data indicate that SLC6A6 overexpression reduced VSMC proliferation and migration and reversed VSMC dedifferentiation induced by PDGF-BB.

### 3.6. SLC6A6 Regulated the Functions of VSMCs via β-Catenin Pathway

Previous studies have shown that SLC6A6 could regulate adipose-derived stem cell differentiation via β-catenin signaling [33], which is a major pathway involved in VSMC dysfunction [34]. Therefore, we hypothesized that SLC6A6 may mediate VSMC regulation through this pathway. We investigated the changes in β-catenin expression after SLC6A6 overexpression or knockdown in VSMCs using Western blotting. The Western blotting analysis revealed that SLC6A6 knockdown resulted in upregulation of β-catenin, whereas SLC6A6 overexpression decreased β-catenin levels (Figure 6 C,D). These data indicate that SLC6A6 exerts its influence on VSMC dedifferentiation, proliferation, and migration, at least in part, via β-catenin signaling.

### 3.7. SLC6A6 Ameliorated Neointimal Formation after Vascular Injury

**Figure 7:**
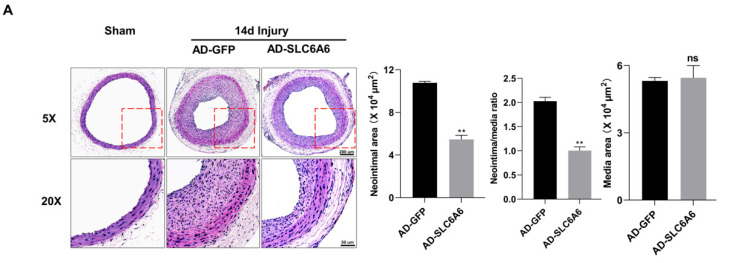

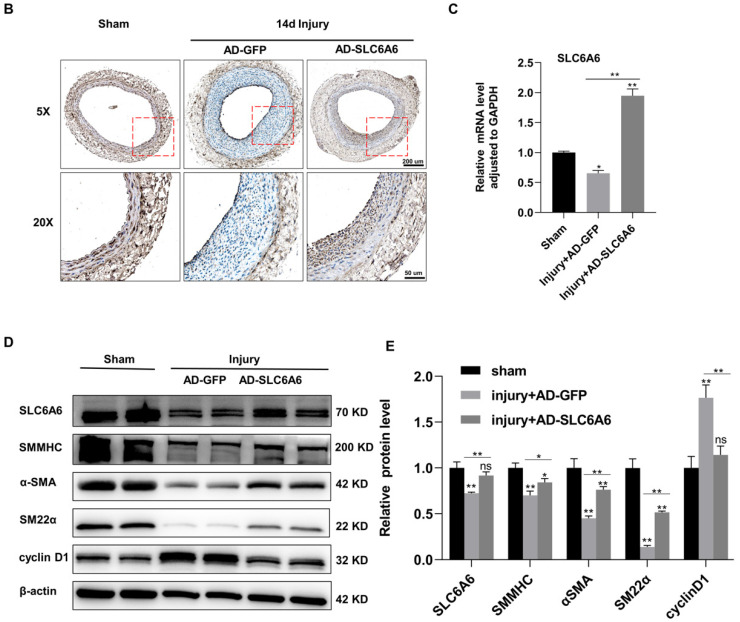
Local SLC6A6 overexpression in vivo ameliorated neointimal formation after vascular injury. (**A**) Representative HE stained in sham group and injured carotid arteries treated with AD-GFP or AD-SLC6A6 for 14 days after injury. Scale bar: 200 µm (50× upper). Scale bar: 50 µm (200× lower). Quantitative analysis of the intima area, media area, and the ratio of intima to media and media area. N = 6; ns, not significant; * p < 0.05, ** *p* < 0.01 vs. AD-GFP; data are represented as mean ± SD. (**B**) Representative cross-sections of immunohistochemically stained SLC6A6 (brown) in carotid arteries treated with AD-GFP or AD-SLC6A6 for 14 days after injury. Scale bar: 200 µm (50× upper). Scale bar: 50 µm (200× lower). (**C**) The mRNA level of SLC6A6 in sham group and injured carotid arteries treated with AD-GFP or AD-SLC6A6 for 14 days after injury. Data are normalized to GAPDH. N = 6; ns, not significant; * p < 0.05, ** *p* < 0.01 vs. sham; data are represented as mean ± SD. (**D**,**E**) Western blot analysis of SLC6A6, contractile VSMC-specific proteins (SMMHC, αSMA, SM22α) and cell proliferation marker cyclin D1 in sham group, and injured carotid arteries treated with AD-GFP or AD-SLC6A6 for 14 days after injury. Data are normalized to β-actin. N = 6; ns, not significant; * p < 0.05, ** *p* < 0.01; label *, **, ns on the bars compares with sham group. Data are represented as mean ± SD.

**Figure 8:**
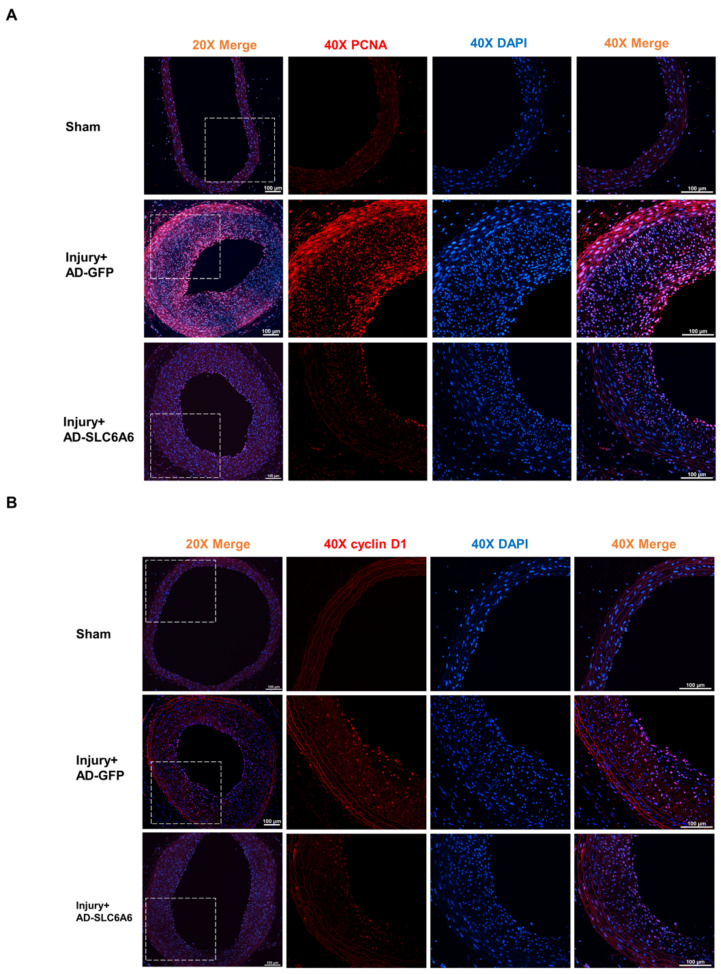
SLC6A6 overexpression reduced VSMC proliferation in vivo. (**A**) Immunofluorescence staining shows PCNA (red) and DAPI (blue) expression and localization in sham group and injured carotid arteries treated with AD-GFP or AD-SLC6A6 for 14 days post injury. Scale bar: 100 µm (200× left); scale bar: 100 µm (400× right). (**B**) Immunofluorescence staining showing cyclin D1 (red) and DAPI (blue) expression and localization in sham group and injured carotid arteries treated with AD-GFP or AD-SLC6A6 for 14 days post injury. Scale bar: 100 µm (200× left); scale bar: 100 µm (400× right).

Given the role of SLC6A6 in VSMCs, we investigated whether SLC6A6 could effectively reduce neointimal formation after vascular injury. As shown in in vitro overexpression experiments, we locally expressed SLC6A6 in vivo by transfecting rats with adenovirus vectors (AD-SLC6A6), and AD-GFP was used as the control. After inducing vascular injury in the common carotid artery of rats, AD-GFP or AD-SLC6A6 was infused to induce local VSMC infection. Fourteen days after injury, H&E staining of the injured tissue revealed that AD-SLC6A6 significantly reduced neointimal formation compared to AD-GFP, and the intima/media ratio in the AD-SLC6A6 group was greatly reduced compared to that in the AD-GFP group (Figure 7A). Immunohistochemistry analysis confirmed successful local SLC6A6 overexpression in vivo (Figure 7B), and qRT-PCR and Western blotting further confirmed that SLC6A6 mRNA and protein levels were increased in the AD-SLC6A6 group compared to controls (Figure 7C,D). As expected, vascular injury downregulated VSMC contractile proteins (SMMHC, α-SMA, and SM22α) but upregulated cell proliferation marker cyclin D1, while SLC6A6 overexpression in vivo reversed these expression changes after vascular injury (Figure 7D,E). Immunofluorescence staining further confirmed that the expression levels of proliferation markers cyclin D1 and PCNA were downregulated in the AD-SLC6A6 group compared to controls (Figure 8A,B). These data demonstrated that SLC6A6 overexpression ameliorated neointimal formation after vascular injury via suppressing VSMC proliferation and dedifferentiation in vivo.

## 4. Discussion

VSMC proliferation, migration, and dedifferentiation are critical cellular events in vascular remolding, such as atherosclerosis and restenosis. In this study, we identified an important role for SLC6A6 in regulating these VSMC functions. Mechanistically, SLC6A6 overexpression inhibits VSMC proliferation, migration, and dedifferentiation, thereby preventing neointimal formation by inhibiting the β-catenin pathway. Our results provide a potential target for the treatment of neointimal formation.

As a consequence of endothelial dysfunction induced by vascular injuries, VSMCs are exposed to platelet aggregation and circulating growth factors, such as PDGF-BB, which promotes VSMC proliferation, migration, and dedifferentiation, leading to neointimal formation [35]. In this study, we revealed that SLC6A6 is downregulated during vascular injury. Consistent with our observations, SLC6A6 expression is decreased in acute kidney injury and ischemic injury of cardiomyocytes, while overexpression of SLC6A6 could ameliorate these conditions [19,36], implying that SLC6A6 has a protective effect against injury. Several studies have reported that oxidative stress and reactive oxygen species (ROS) downregulate SLC6A6 [19,36,37,38], while ROS levels are increased in atherosclerosis, vascular injury [39], and VSMCs treated with PDGF-BB (Figure S1). These reports suggest that accumulation of ROS may be responsible for the downregulation of SLC6A6 observed in vascular injury and atherosclerosis and dedifferentiated VSMCs.

Multiple studies have confirmed the protective effect of taurine on the cardiovascular system [24,27,28,40]. Taurine can suppress VSMC proliferation both in vitro and in vivo [26,41]. As a taurine transporter, SLC6A6 maintains high intracellular taurine concentrations, and SLC6A6 knockdown reduces intracellular taurine, whereas SLC6A6 upregulation increases taurine uptake by VSMCs [9,10,42]. Notably, the concentration of taurine in the aorta is 40 times higher than that in plasma [43]. In this study, tissue immunofluorescence demonstrated that SLC6A6 expression is localized to the medial layer and adventitia of the normal vasculature, while almost not in the neointimal layer after vascular injury and atherosclerosis (Figure 2E and Figure 3C). This observation is in line with previous studies reporting that taurine is mainly located in the vascular adventitia, but not the neointimal layer, after injury [26].

Wnt/β-catenin signaling, a critical pathway in vascular diseases, promotes VSMC proliferation, migration, and dedifferentiation [44,45,46]. This pathway is activated when VSMCs are stimulated by growth factors or vascular injury. Activated β-catenin is translocated to the nucleus to bind to the TCF/LEF family of transcription factors, and it regulates the transcription of target genes, such as cyclin D1 and matrix metalloproteinases et al. [34,47]. In this study, SLC6A6 overexpression resulted in the inhibition of β-catenin signaling and downregulation of cyclin D1, thereby preventing VSMC proliferation. Additionally, we found that SLC6A6 overexpression in vivo significantly suppressed neointimal formation and the expression of cyclin D1 and PCNA, which are cell proliferation markers (Figure 8). Furthermore, Liao et al. reported that L-carnitine and taurine inhibited VSMC proliferation by increasing the expression of SLC6A6 [9,29], which further supports our results. The correlation between SLC6A6 and β-catenin pathway activation is consistent with previous studies showing that SLC6A6 knockdown prevents the reduction in β-catenin levels during human adipose-derived stem cell differentiation [33] and that β-catenin is activated in the SLC6A6-knockout mice [15].

During VSMC dedifferentiation, cells downregulate the expression of contractile phenotype genes and transition to a synthetic phenotype characterized by higher proliferative and migratory abilities. In this study, we discovered that SLC6A6 expression was positively correlated with contractile VSMC-specific protein expression both in vitro and in vivo. SLC6A6 overexpression prevented the downregulation of these proteins induced by PDGF-BB or vascular injury (Figure 6A and Figure 7D). These results imply that SLC6A6 is involved in VSMC differentiation. This is consistent with other research showing that SLC6A6 regulates the differentiation of skeletal muscle cells [48] and human adipose-derived stem cells [33], reprograms the polarized phenotype of macrophages, and regulates trophoblast differentiation via SLC6A6-mediated taurine influx [10,49]. In this study, overexpression of SLC6A6 reduced the ROS level induced by PDGF-BB (Figure S1). Recent studies have confirmed that ROS level is an important factor leading to phenotypic switching of VSMCs [50], which may be one of the reasons why overexpression of SLC6A6 can reverse VSMC dedifferentiation. Interestingly, the protein–protein interaction network analysis using the STRING database (https://cn.string-db.org) revealed that there may be a co-expression relationship between SLC6A6 and MYH11 (SMHHC) (Figure S2), and our experiments confirmed that SMMHC expression was upregulated upon SLC6A6 overexpression. However, the mechanisms of SLC6A6 in VSMC differentiation are not clear, which requires further investigation.

There are some limitations in this study. Firstly, the number of clinical samples in this experiment is limited, and more clinical samples would increase the practicality of this study. Secondly, this study demonstrated that SLC6A6 is closely related to the phenotypic switching of VSMCs, and the mechanism needs further study. To further clarify its regulatory mechanism, protein mass spectrometry, co-immunoprecipitation, and other experimental methods may be used in the future. Finally, this study focused on the regulation of SLC6A6 on VSMC function, but it is not clear whether SLC6A6 could affect the function of vascular endothelial cells, which also plays an important role in intimal hyperplasia. This will be another research direction in the future.

In summary, this study reports a novel role of SLC6A6 in inhibiting VSMC proliferation and migration and maintaining the contractile phenotype of VSMCs. Importantly, overexpression of SLC6A6 reduced neointimal formation after vascular injury. These effects of SLC6A6 are mediated, at least in part, by reducing ROS production and blocking the Wnt/β-catenin pathway (graphical abstract). Therefore, we propose that SLC6A6 may be a promising target for decreasing neointimal formation in atherosclerosis patients.

## Data Availability

The data presented in this study are available from the first author upon reasonable request.

## Supplementary Materials

The following supporting information can be downloaded at: https://www.mdpi.com/article/10.3390/ijms24033018/s1.
