# Supplementary material for: Ant-Neointimal Formation Effects of SLC6A6 in Preventing Vascular Smooth Muscle Cell Proliferation and Migration via Wnt/β-Catenin Signaling
Source: Int J Mol Sci. 2023 Feb 3;24(3):3018. doi: 10.3390/ijms24033018 (PMC9917619; doi:10.3390/ijms24033018)
Supplement: Supplementary file 1 [file ijms-24-03018-s001.zip › ijms-2143493-supplementary.pdf]

SLC6A6 suppresses vascular smooth muscle cell proliferation, migration and neointima formation via Wnt/β-catenin signaling

Supplement

Supplementary Figure 1

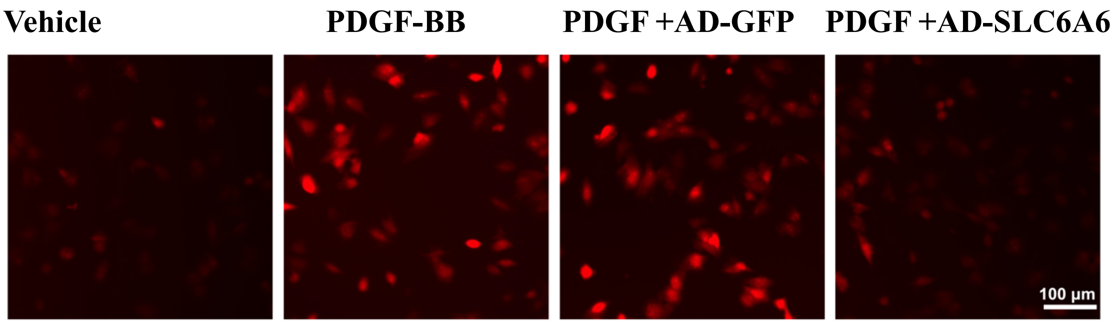

SLC6A6 prevented PDGF-BB-induced ROS production in VSMCs. ROS generation was evaluated by dhydroethidium (DHE) fluorescence (Red). scale bar:100 μm.

Supplementary Figure 2

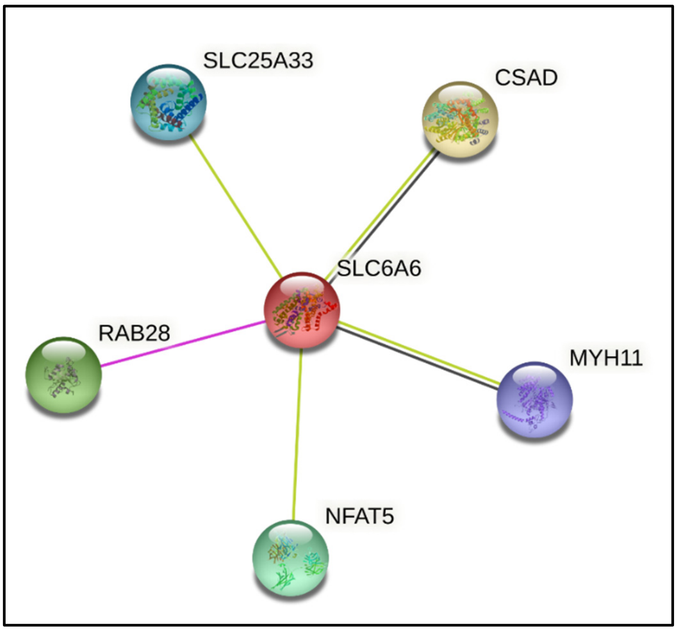

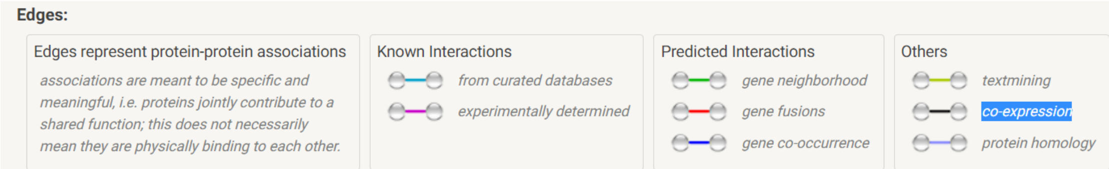

SLC6A6 Protein-protein interaction network is downloaded in the web https://cn.string-db.org/

Supplementary Table 1: PCR Primer Sequences

Name	sequences
**RAT SLC6A6 forward primer**	**5' GTTCGGTCCACAGGCAAGGTTG 3'**
**RAT SLC6A6 reverse primer**	**5' GTCCTCAAGGCGGCTGATGTTAG 3'**
**RAT α-SMA forward primer**	**5' CTGCCTTGGTGTGTGACAATGG 3'**
**RAT α-SMA reverse primer**	**5' CGGGTACTTCAGGGTCAGGATTC 3'**
**RAT SMMHC forward primer**	**5' GCCGCTGCCTATGACAAACT 3'**
**RAT SMMHC reverse primer**	**5' CGCTGGTTGTCCAAGTCCA 3'**
**RAT cyclinD1 forward primer**	**5' CTACCGCACAACGCACTTTC 3'**
**RAT cyclinD1 reverse primer**	**5' TTCCTCCACTTCCCCTTCCT 3'**
**RAT GAPDH forward primer**	**5' ACCCAGAAGACTGTGGATGG 3'**
**RAT GAPDH reverse primer**	**5' CACATTGGGGGTAGGAACAC 3'**
**Homo SLC6A6 forward primer**	**5' TGGGTAGGCAATGAAGGCCAG 3'**
**Homo SLC6A6 reverse primer**	**5' TTTTGTGTCTGGCTTCGCAATTT 3'**
**Homo GAPDH forward primer**	**5' ACAACTTTGGTATCGTGGAAGG 3'**
**Homo GAPDH reverse primer**	**5' GCCATCACGCCACAGTTTC 3'**

Supplementary Table 2: Baseline Patient Characteristics

**Patient Characteristic**	**Healthy control group (n=4)**	**Atherosclerosis artery group (n=4)**	**P value**
**Demographics**			
Female sex， no.(%)	2 (50%)	1 (25%)	0.465#
Age (years)	40.5±4.8	72±5.6	0.569*
**Clinical Parameters**			
Smoker, no. (%)	1 (25%)	3 (75%)	0.157#
BMI, kg/m2	23.25±3.4	23.25±0.9	1 *
SBP, mmHg	118.75±2.22	134.5±11.47	0.066*
DBP, mmHg	80±2.16	87.25±2.75	0.379*
**Comorbidities**			
Carotid artery disease, no. (%)	0 (0)	3 (75%)	NA

Coronary artery disease, no. (%)	0 (0)	3 (75%)	NA
Hypertension, no. (%)	0 (0)	3 (75%)	NA
Type II diabetes mellitus, no. (%)	0 (0)	2 (50%)	NA
Hyperlipidemia, no. (%)	0 (0)	3 (75%)	NA

N=4 *P* value is calculated with t-test (*), Fisher's exact test (#). Carotid/coronary artery disease was defined as the arterial lumen stenosis rate greater than 70%. BMI, body mass index; SBP, systolic blood pressure; DBP, diastolic blood pressure.
